# The Safety and Effectiveness of Bevacizumab in the Treatment of Nonsquamous Non-Small-Cell Lung Cancer: A Meta-Analysis of Randomized Controlled Trials

**DOI:** 10.1155/2021/5537899

**Published:** 2021-09-07

**Authors:** Yue Zhou, Mei He, Rui Li, Yuan Peng, Feng Li, Shengqian Li, Ming Yang

**Affiliations:** Department of Pharmacy, Affiliated Hospital of North Sichuan Medical College, China

## Abstract

**Objective:**

Bevacizumab was currently available for nonsquamous non-small-cell lung cancer (NSqNSCLC) patients and has been studied in several randomized controlled trials (RCTs) for treatment of these patients. This meta-analysis summarizes the most up-to-date evidences regarding the effects and adverse reactions of bevacizumab in the treatment of NSqNSCLC patients.

**Methods:**

The authors searched for RCTs from electronic database including PubMed, EMBASE, and the Cochrane Central Register of Controlled Trials. Experimental arm was defined as the bevacizumab-containing group and the control arm as the bevacizumab-free group. Data of objective response rate (ORR), disease control rate (DCR), progression-free survival (PFS), overall survival (OS), and adverse reactions were synthetically extracted. A protocol for this meta-analysis has been registered on PROSPERO (http://www.crd.york.ac.uk/prospero).

**Results:**

Ten RCTs that involved a total of 3134 patients were included. The experimental group was associated with significant superior ORR (RR 1.63, 95% CI 1.24 to 2.14, *P* < 0.001), OS (HR 0.90, 95% CI 0.82 to 0.99, *P* < 0.001), and prolonged PFS (HR 0.68, 95% CI 0.62 to 0.74, *P* < 0.001) compared to the control. No significant difference was observed regarding DCR (RR 1.13, 95% CI 0.99 to 1.30, *P* = 0.08). The experimental group showed higher rate of hypertension (RR 6.91, 95% CI 4.62 to 10.35, *P* < 0.00001) and hemorrhagic events (RR 3.07, 95% CI 1.78 to 5.30, *P* < 0.0001) than the control group. The experimental group showed lower rate of anemia (RR 0.72, 95% CI 0.55 to 0.96, *P* = 0.02) than the control group. No significant difference was observed regarding treatment-related adverse event grade 3-5 (TRAE3-5) (RR 1.23, 95% CI 0.99 to 1.53, *P* = 0.06), thrombocytopenia (RR 1.11, 95% CI 0.92 to 1.33, *P* = 0.29), and neutropenia (RR 1.11, 95% CI 0.88 to 1.40, *P* = 0.36).

**Conclusion:**

This meta-analysis showed that bevacizumab could increase ORR, OS, and prolonged PFS for treatment of NSqNSCLC patients. However, no significant improvement in DCR was observed and bevacizumab could increase the rate of hypertension and hemorrhagic events. Bevacizumab was an acceptable option for NSqNSCLC patients. This trial is registered with *PROSPERO registration number*: CRD42021226790.

## 1. Introduction

As the leading cause of cancer death worldwide, lung cancer accounts for 18.4% of the total cancer deaths [1]. Non-small-cell lung cancer (NSCLC) accounts for approximately 80–85% of all lung cancer cases and usually allocated to advanced stage at their first diagnosis [2]. The NCCN guideline suggested that systemic palliative chemotherapy and/or radiotherapy remained the standard care for these locally advanced or metastatic NSCLC patients [3].

To inhibit angiogenesis is another treatment option, because tumor angiogenesis is critical for the process of primary tumor growth, proliferation, differentiation, and metastasis and has been identified as an important therapeutic target for tumor in recent decades [4, 5]. Antiangiogenic therapy has been used for cancer treatment, which inhibits the delivery of oxygen and nutrients to cancer cells [6]. As a key mediator of angiogenesis, vascular endothelial growth factor (VEGF) and its receptors are considered to be the pivotal pathway in angiogenesis-related molecular mechanisms which have been well studied [7, 8].

Bevacizumab is a VEGF monoclonal antibody, which inhibits angiogenesis to suppress tumor growth by restricting oxygen and nutrient supply to tumors [9]. Increasing numbers of clinical trials have been conducted with bevacizumab for the treatment of patients with advanced NSCLC since it was approved. Previous meta-analysis found that bevacizumab used in combination with paclitaxel and carboplatin did increase objective response rate (ORR), overall survival (OS), and prolonged progression-free survival (PFS) compared with paclitaxel and carboplatin for NSCLC [10–14]. However, the important outcome disease control rate (DCR) was not analyzed and only five RCTs were included. Whether bevacizumab containing could improve ORR, DCR, and OS and increase adverse reactions for treatment of nonsquamous non-small-cell lung cancer (NSqNSCLC) patients is still controversial. In consideration of these controversial results, we carried out this meta-analysis. Our meta-analysis includes 10 randomized controlled trials (RCTs) and identifies the precise effect of bevacizumab containing for NSqNSCLC patients on outcomes of ORR, DCR, OS, PFS, and treatment-related adverse event compared with bevacizumab free.

## 2. Method

### 2.1. Protocol and Registration

This meta-analysis was performed according to the PRISMA (Preferred Reporting Items for Systematic Reviews and Meta-Analyses) recommendations. This study was not a human or animal experiment; thus, ethical approval was not necessary. A protocol for this meta-analysis has been registered on PROSPERO (http://www.crd.york.ac.uk/prospero), and the registration number is CRD42021226790.

### 2.2. Search Strategy

Databases including PubMed, EMBASE, and the Cochrane Central Register of Controlled Trials were searched with a combination of the terms “non-small-cell lung cancer (NSCLC) or nonsquamous non-small-cell lung cancer (NSqNSCLC)” and “angiogenesis inhibitors or bevacizumab” within the restriction limit of “randomized controlled trial (RCT).” In addition, reference lists of the included studies were manually checked for potentially eligible studies and Google Scholar search engines were used to find additional references. The last search was performed on December 8, 2020, without any restriction to language of publication.

### 2.3. Inclusion and Exclusion Criteria

Inclusion criteria are as follows: (1) research types: RCTs publicly published at home and abroad; (2) research objects: adult patients with confirmed locally advanced or metastatic NSqNSCLC; and (3) intervention measures: the experimental group using bevacizumab plus standard chemotherapy regimen and the control group using standard chemotherapy regimen alone. Exclusion criteria are as follows: exclude articles that do not meet the inclusion criteria, cannot obtain the main indicators in the article, and have not received a response through contacting the author, and republished articles.

### 2.4. Quality Assessment and Data Extraction

The Cochrane risk of bias tool was used to evaluate the quality of each study by two reviewers, and the following 7 categories were assessed: random sequence generation, allocation concealment, blinding of participants and personnel, blinding of the outcome assessment, incomplete outcome data, selective outcome reporting, and other biases [15]. The overall methodologic quality of each included study was assessed as “low risk of bias,” “high risk of bias,” or “unclear risk of bias.” A third reviewer would be invited if there were any dispute.

Two authors independently completed the data extraction. The extracted general data included author, year, and country of publication and sample size. The primary end-point was OS, and the secondary end-points contained ORR, DCR, PFS, grade 3-5 of treatment-related adverse event (TRAE3-5), hypertension neutropenia, thrombocytopenia, anemia, and hemorrhagic events.

### 2.5. Statistical Analysis

Outcomes were estimated by calculating the pooled risk ratio (RR) (95% confidence intervals [CIs]) for ORR, DCR, and TRAE by RevMan software (version 5.1; Cochrane Collaboration, Copenhagen, Denmark), and hazard ratio was pooled for survival outcomes (OS and PFS) by STATA version 12.0 (StataCorp, College Station, TX). A *P* < 0.05 was considered statistically significant. Heterogeneity was assessed by visual inspection of the forest plot combined with the results of the test for heterogeneity and the *I*^2^ test. Fixed-effects model would be employed for outcomes with low heterogeneity (*I*^2^ < 50%). Otherwise, the random-effects model of DerSimonian and Laird [16] would be selected. Sensitivity analysis would be conducted by omission of each single study to evaluate stability of the results if heterogeneous studies existed.

## 3. Results

### 3.1. Search Results and Characteristics of Included Studies

**Figure 1:**
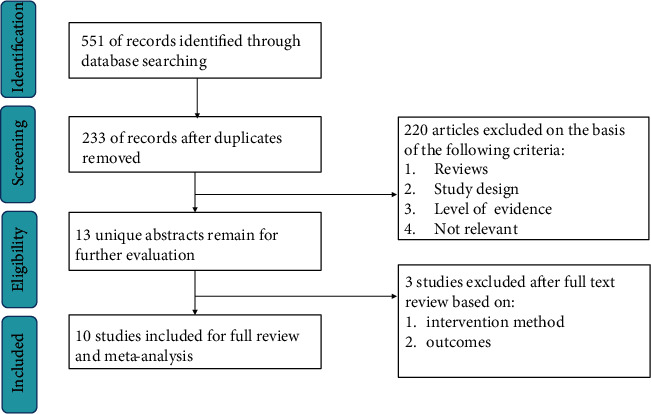
Flow diagram shows the process of literature selection.

551 potential articles were initially identified through database searches on 8 December 2020. Two hundred and thirty-three studies were considered potentially eligible for further assessment after duplicates were removed. Finally, 10 RCTs [17–26] that involved a total of 3134 patients published between 2006 and 2020 met the inclusion criteria and were included in this meta-analysis after a full-text review.  Figure 1 shows the literature selection process.  Table 1 summarizes the details of both the included studies and agents.

### 3.2. Risk of Bias

**Figure 2:**
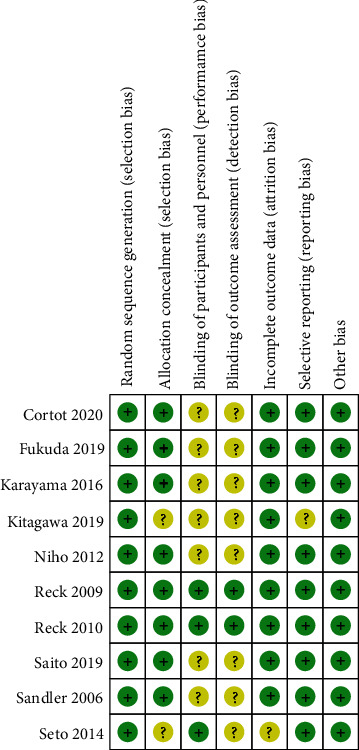
The methodological quality of the RCTs.

All included RCTs were assessed by two authors independently according to Cochrane risk of bias tool. Detailed information can be found in Figure 2.

### 3.3. Outcomes of the Bevacizumab-Containing Group versus the Bevacizumab-Free Group

#### 3.3.1. Efficacy Profile

**Figure 3:**
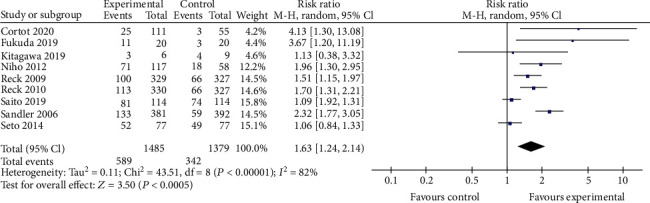
ORR.

**Figure 4:**
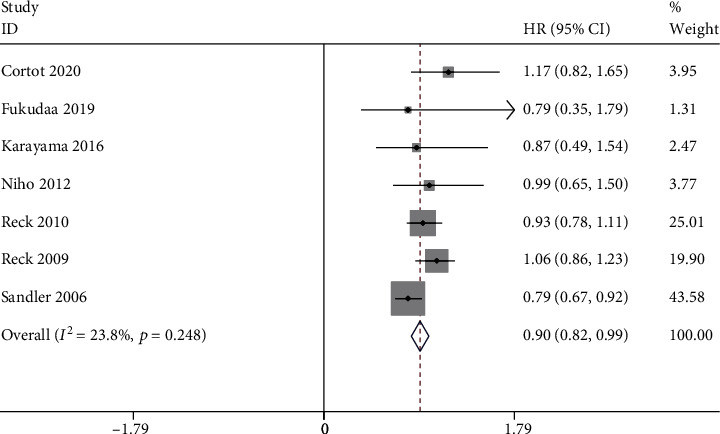
OS.

**Figure 5:**
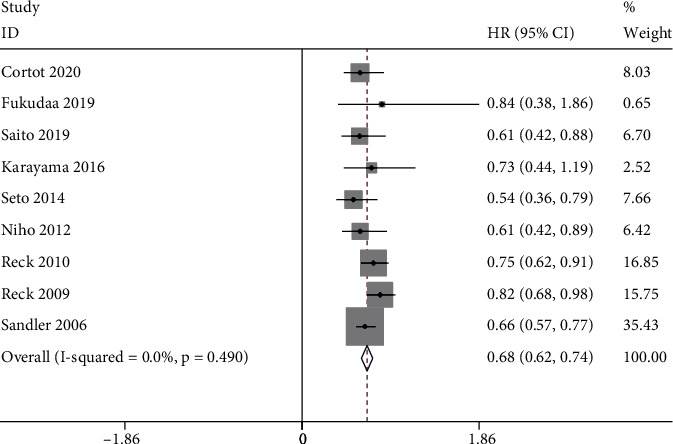
PFS.

**Figure 6:**
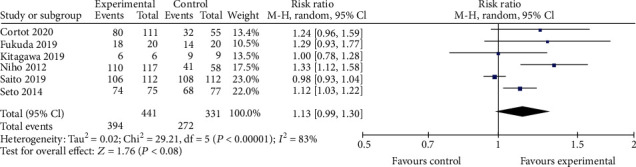
DCR.

Compared to the bevacizumab-free group, the bevacizumab-containing group was associated with significantly superior ORR (RR 1.63, 95% CI 1.24 to 2.14, *P* < 0.001; Figure 3), OS (HR 0.90, 95% CI 0.82 to 0.99, *z* = 21.45, *P* < 0.001; Figure 4), and longer PFS (HR 0.68, 95% CI 0.62 to 0.74, *z* = 22.50, *P* < 0.001; Figure 5). However, no significant improvement in DCR (RR 1.13, 95% CI 0.99 to 1.30, *P* = 0.08; Figure 6) was observed.

#### 3.3.2. Safety Profile

**Figure 7:**
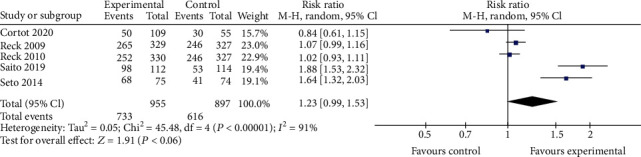
TRAE3-5.

**Figure 8:**
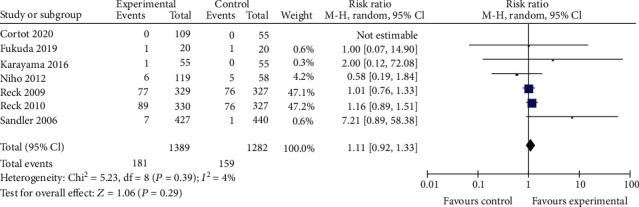
Thrombocytopenia.

**Figure 9:**
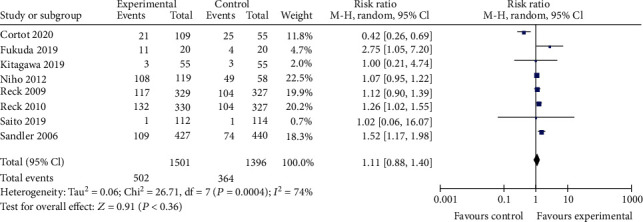
Neutropenia.

**Figure 10:**
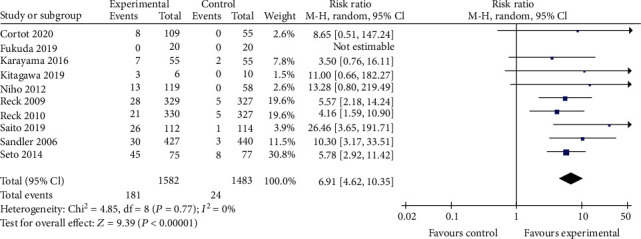
Hypertension.

**Figure 11:**
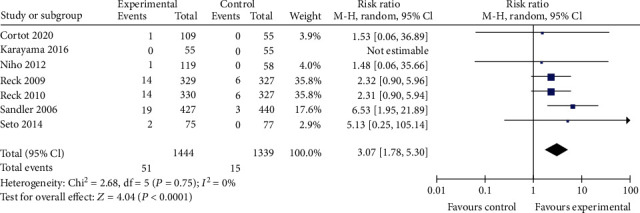
Hemorrhagic event.

**Figure 12:**
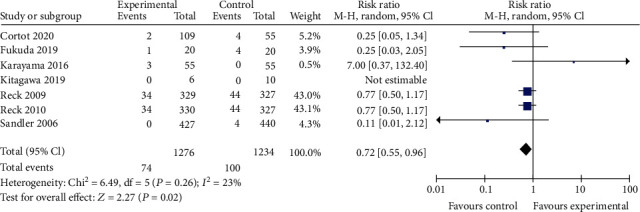
Anemia.

For TRAE3-5, thrombocytopenia, and neutropenia outcomes, no significant difference was observed between the bevacizumab-containing group and bevacizumab-free group (RR 1.23, 95% CI 0.99 to 1.53, *P* = 0.06; RR 1.11, 95% CI 0.92 to 1.33, *P* = 0.29; and RR 1.11, 95% CI 0.88 to 1.40, *P* = 0.36 Figures 7–9). The bevacizumab-containing group showed higher rate of hypertension and hemorrhagic events than the bevacizumab-free group (RR 6.91, 95% CI 4.62 to 10.35, *P* < 0.00001 and RR 3.07, 95% CI 1.78 to 5.30, *P* < 0.0001; Figures 10 and 11). The bevacizumab-containing group showed lower rate of anemia than the bevacizumab-free group (RR 0.72, 95% CI 0.55 to 0.96, *P* = 0.02; Figure 12).

#### 3.3.3. Sensitivity Analysis

Sensitivity analysis indicated that omitting any single study did not significantly affect the pooled RR for ORR (Table 2). For DCR, omitting Saito (2019) showed that *I*^2^ was decreased to 45% and significant difference was observed (RR 1.18, 95% CI 1.05 to 1.32, *P* < 0.01; Table 3). For TRAE3-5, omitting Cortot (2020) showed that *I*^2^ was 93% and significant difference was observed (RR 1.32, 95% CI 1.04 to 1.68, *P* < 0.05; Table 4). For neutropenia, omitting Cortot (2020) showed that *I*^2^ was decreased to 48% and significant difference was observed (RR 1.23, 95% CI 1.05 to 1.44, *P* = 0.01; Table 5).

## 4. Discussion

Interference with VEGFR functions has been an alternative approach for the treatment of NSCLC [27]. Bevacizumab, a novel targeted therapeutic, differs in their modes of action and tolerability profiles from those of cytotoxic agents and can be combined with traditional chemotherapy to offer greater clinical benefits [25]. It has been approved for use in combination with the standard platinum-based chemotherapy or as a maintenance therapy after chemotherapy during the treatment of NSCLC patients without driver mutations [26, 28]. However, little information was reported on DCR and TRAE of NSqNSCLC patients. Besides, there have been several novel studies published afterwards. Thus, it is necessary to update the results.

We conducted this meta-analysis with 10 RCTs included 3134 advanced NSqNSCLC patients to compare therapeutic efficacy and adverse reactions of bevacizumab containing and bevacizumab free for NSqNSCLC patients. According to the current outcomes, treatment regimens containing bevacizumab had significant improvements for ORR, OS, and PFS outcomes, when compared with the treatment regimens without bevacizumab. However, significant outcome was not observed in DCR. It is indicated that significant improvements of ORR and PFS could translate into overall survival benefits. A previous meta-analysis reported similar results regarding efficacy profile [10]. However, the important outcome disease control rate (DCR) was not analyzed and only five RCTs were included. Five RCTs including 1852 patients, 7 RCTs including 2671 patients, and 8 RCTs including 2897 patients indicated that the bevacizumab-containing group and bevacizumab-free group had a similar rate of TRAE3-5, thrombocytopenia, and neutropenia. All 10 RCTs reported hypertension and 7 RCTs reported hemorrhagic events, and the bevacizumab-containing group showed a higher rate of hypertension and hemorrhagic events compared to the bevacizumab-free group. Seven RCTs reported anemia and the bevacizumab-containing group showed a lower rate of anemia compared to the bevacizumab-free group.

Though more RCTs were included in this meta-analysis, improvement was not observed with respect to DCR in the bevacizumab-containing group. A possible reason is that Saito (2019) affected the result, because sensitivity analysis indicated that omitting Saito (2019) showed that *I*^2^ was decreased to 45% and significant difference was observed for DCR. Other possible reasons include that bevacizumab may not improve DCR or limited number of RCTs limits the positive result. More multicenter, large-sample RCTs or even real-world studies comparing bevacizumab for NSqNSCLC patients are urged to validate the DCR as DCR was considered as one of the important outcomes for cancer patients.

## 5. Limitations of This Study

First, we did not perform subgroup analysis as subgroup analysis will result in limited articles available. Second, various chemotherapeutic regimens and patterns are involved in different RCTs. This may lead to a certain degree of heterogeneity and significant heterogeneities. Third, we cannot extract more data to complete an in-depth analysis of DCR and more high-quality trials are warranted to support the survival benefit of bevacizumab.

## 6. Conclusions

Our meta-analysis showed that treatment containing bevacizumab was an option for patients with NSqNSCLC and patients with acceptable efficacy. Bevacizumab was superior to those without it in terms of ORR, OS, and PFS in patients with NSqNSCLC and no significant TRAE3-5 was observed.

## Figures and Tables

**Table 1:** Characteristics of included studies.

Studies	Year	Intervention		Number (case/control)	Type of study
		Experimental group	Control group		
Cortot et al.	2020	Bevacizumab+paclitaxel	Docetaxel	111/55	RCT
Kitagawa et al.	2019	Bevacizumab+gefitinib	Gefitinib	6/10	RCT
Fukuda et al.	2019	Bevacizumab+pemetrexed	Pemetrexed	20/20	RCT
Saito et al.	2019	Bevacizumab+erlotinib	Erlotinib	114/114	RCT
Karayama et al.	2016	Bevacizumab+pemetrexed	Pemetrexed	55/55	RCT
Seto et al.	2014	Bevacizumab+erlotinib	Erlotinib	77/77	RCT
Niho et al.	2012	Bevacizumab+carboplatin+paclitaxel	Carboplatin+paclitaxel	121/59	RCT
Reck et al.	2010	Bevacizumab 7.5 mg/kg+cisplatin+gemcitabine	Placebo+cisplatin+gemcitabine	345/347	RCT
Reck et al.	2009	Bevacizumab 15 mg/kg+cisplatin+gemcitabine	Placebo+cisplatin+gemcitabine	351/347	RCT
Sandler et al.	2006	Bevacizumab+paclitaxel+carboplatin	Paclitaxel+carboplatin	417/433	RCT

RCT: randomized controlled trial.

**Table 2:** Sensitivity analyses based on various exclusion criteria for ORR.

Excluded trial	No. of trials	No. of patients	Experimental group	Control group	RR (95% CI)	*P* value for RR	*I*^2^(%)	*P* value for heterogeneity
Cortot (2020)	8	2698	1374	1324	1.56 [1.19, 2.04]	<0.01	82	<0.01
Fukuda (2019)	8	2824	1465	1359	1.56 [1.19, 2.06]	<0.01	83	<0.01
Kitagawa (2019)	8	2849	1479	1370	1.66 [1.25, 2.20]	<0.01	84	<0.01
Niho (2012)	8	2689	1368	1321	1.59 [1.18, 2.14]	<0.01	83	<0.01
Reck (2009)	8	2208	1156	1052	1.67 [1.21, 2.31]	<0.01	84	<0.01
Reck (2010)	8	2207	1155	1052	1.63 [1.19, 2.24]	<0.01	83	<0.01
Saito (2019)	8	2636	1371	1265	1.74 [1.31, 2.31]	<0.01	75	<0.01
Sandler (2006)	8	2091	1104	987	1.50 [1.16, 1.93]	<0.01	74	<0.01
Seto (2014)	8	2710	1408	1302	1.75 [1.31, 2.36]	<0.01	79	<0.01

**Table 3:** Sensitivity analyses based on various exclusion criteria for DCR.

Excluded trial	No. of trials	No. of patients	Experimental group	Control group	RR (95% CI)	*P* value for RR	*I*^2^ (%)	*P* value for heterogeneity
Cortot (2020)	5	554	314	240	1.12 [0.97, 1.29]	>0.05	83	<0.01
Fukuda (2019)	5	732	421	311	1.12 [0.97, 1.29]	>0.05	85	<0.01
Kitagawa (2019)	5	757	435	322	1.16 [0.99, 1.35]	>0.05	86	<0.01
Niho (2012)	5	597	324	273	1.09 [0.96, 1.23]	>0.05	73	<0.01
Saito (2019)	5	548	329	219	1.18 [1.05, 1.32]	<0.01	45	<0.01
Seto (2014)	5	620	366	254	1.15 [0.92, 1.43]	>0.05	87	<0.01

**Table 4:** Sensitivity analyses based on various exclusion criteria for TRAE3-5.

Excluded trial	No. of trials	No. of patients	Experimental group	Control group	RR (95% CI)	*P* value for RR	*I*^2^(%)	*P* value for heterogeneity
Cortot (2020)	4	554	846	842	1.32 [1.04, 1.68]	<0.05	93	<0.01
Reck (2009)	4	732	626	570	1.28 [0.89, 1.84]	>0.05	93	<0.01
Reck (2010)	4	757	625	570	1.30 [0.93, 1.82]	>0.05	92	<0.01
Saito (2019)	4	597	635	563	1.11 [0.94, 1.32]	>0.05	84	<0.01
Seto (2014)	4	548	880	823	1.15 [0.93, 1.43]	>0.05	91	<0.01

**Table 5:** Sensitivity analyses based on various exclusion criteria for neutropenia.

Excluded trial	No. of trials	No. of patients	Experimental group	Control group	RR (95% CI)	*P* value for RR	*I*^2^ (%)	*P* value for heterogeneity
Cortot (2020)	7	2733	1392	1341	1.25 [1.12, 1.39]	<0.0001	48	0.07
Fukuda (2019)	7	2857	1481	1376	1.07 [0.85, 1.34]	0.56	74	0.0008
Kitagawa (2019)	7	2789	1446	1341	1.12 [0.88, 1.42]	0.37	78	0.0002
Niho (2012)	7	2720	1382	1338	1.12 [0.81, 1.55]	0.50	76	0.0004
Reck (2009)	7	2241	1172	1069	1.11 [0.82, 1.50]	0.51	78	0.0001
Reck (2010)	7	2240	1171	1069	1.08 [0.80, 1.45]	0.62	76	0.0003
Saito (2019)	7	2671	1389	1282	1.11 [0.88, 1.41]	0.37	78	0.0002
Sandler (2006)	7	2030	1074	956	1.04 [0.81, 1.33]	0.76	71	0.002

## Data Availability

This is a meta-analysis and all relevant data have been displayed in the manuscript.
